# Using Network Dynamical Influence to Drive Consensus

**DOI:** 10.1038/srep26318

**Published:** 2016-05-23

**Authors:** Giuliano Punzo, George F. Young, Malcolm Macdonald, Naomi E. Leonard

**Affiliations:** 1 Department of Mechanical and Aerospace Engineering, Advanced Space Concept Laboratory, University of Strathclyde, Glasgow, UK; 2 Department of Civil and Structural Engineering, The University of Sheffield, Sheffield, UK; 3 Department of Mechanical and Aerospace Engineering, Princeton University, Princeton, NJ, USA

## Abstract

Consensus and decision-making are often analysed in the context of networks, with many studies focusing attention on ranking the nodes of a network depending on their relative importance to information routing. Dynamical influence ranks the nodes with respect to their ability to influence the evolution of the associated network dynamical system. In this study it is shown that dynamical influence not only ranks the nodes, but also provides a naturally optimised distribution of effort to steer a network from one state to another. An example is provided where the “steering” refers to the physical change in velocity of self-propelled agents interacting through a network. Distinct from other works on this subject, this study looks at directed and hence more general graphs. The findings are presented with a theoretical angle, without targeting particular applications or networked systems; however, the framework and results offer parallels with biological flocks and swarms and opportunities for design of technological networks.

The investigation of collective behaviours and dynamics is often carried out through networks. A network is a powerful means to model the interactions in biological and social groups where individuals are represented by nodes and their interactions by edges. The information routing amongst social individuals is responsible for group behaviour and can in some cases explain complex phenomena. The *Trafalgar Effect* in marine insects and the *Chorus Line Hypothesis* in bird flocks are two biological examples of this kind^1^,^2^, where the information of a predator attacking is quickly and efficiently moved across the whole group, driving a coherent group response. The STARFLAG project represents one of the most dramatic examples of how mapping interactions in a biological group provides the basis for understanding their complexity^3^,^4^,^5^. In a time where physical, cultural and social distances are often bridged by the internet, social sciences can also be abstracted from networks^6^. Engineering is also more often facing the challenges of complex group dynamics, as opposed to complicated systems (see examples like the space system proposed by O’Neil and Weigel^7^ or the discontinued DARPA F6 project^8^ aimed at exploring highly responsive satellite formations). As such, understanding and leveraging the fundamental mechanisms of consensus have become a priority. Algorithms, analytic proofs and empirical studies have been widely reported, shedding new light on this matter^9^,^10^,^11^,^12^,^13^,^14^,^15^,^16^.

It is intuitively clear why the identification of the most influential nodes in a network, those that have a key role in leading and routing information, is of fundamental importance. As such, investigations have been conducted across different scientific areas^17^,^18^,^19^,^20^ with several measurements of node centrality being identified. Node Degree^21^,^22^ considers how many links are associated with a single node, while Betweenness Centrality^23^ ranks the nodes based on the number of geodesic paths (paths of minimum length between any two nodes) that pass through the node. Eigenvector Centrality identifies the nodes best placed in the network as either having many connections or connected to nodes that have many connections^24^. An interesting application of Eigenvector Centrality was proposed by Allesina as a means to measure the species co-extinction through food webs^25^. Estrada considered several centrality measures to investigate the role of peer pressure^26^, obtaining comparable results but showing how different measures of influence highlight alternative aspects of networks.

To understand the role of eigenvector-based analysis it is useful to describe the network as a matrix with binary entries, where nodes are indexed along the rows and columns. If there is a link between any two nodes, say *i* and *j*, meaning that node *i* observes node *j*, then the corresponding entry of the matrix is “1”, otherwise, it is “0”. Such a matrix is termed the adjacency matrix. The spectral properties of the adjacency matrix are fundamental to Eigenvector Centrality as well as other popular ranking methods such as Katz’s centrality^27^ and PageRank^28^.

Consider a general dynamical system based on *N* interconnected nodes. These nodes pursue a final state by observing their neighbours and trying to minimise the relative differences. Such group dynamics are described through


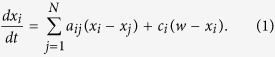


Here *a*_*ij*_ is the *ij* entry of the adjacency matrix, where *x*_*i*_ is the state of node *i*, and its evolution is described by the dynamical system. *w* is a target state that nodes perceive in terms of the difference with respect to their own state and *c*_*i*_ is a gain reflecting the influence of the external driver towards the target state. If the system had no constraints, then a fast consensus to the target would be achieved by increasing the gain *c*_*i*_ as much as possible, up to the point in which the social term of the agent dynamics could be neglected compared to the push towards the external signal. However, when *c*_*i*_ is constrained, as in the present work, then *c*_*i*_ should be cleverly assigned. This elementary form of network dynamical system can be put in vector form as


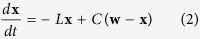


where *L* is known as the Laplacian matrix and, by construction, has elements in all rows summing to 0. *C* is a diagonal matrix of size *N* containing the *c*_*i*_ gains, and **w** is a vector with identical elements representing the target state of the system. The zero row sum of the Laplacian matrix implies 0 is an eigenvalue of *L*, and its first left eigenvector (FLE) can be used to rank the nodes based on their ability to serve as initiators of some dynamics expressed through the Laplacian^29^,^30^. Such a measure was named *Dynamical Influence*^31^ and obtained with particular reference to the ability of a node to serve as initiator of some outbreak. Dynamical influence looks at the first left eigenvector of the Laplacian as opposed to Eigenvector Centrality, which concentrates on the FLE of the adjacency matrix.

A classification of the nodes in the dynamical system context can consider several parameters. One is the ability to achieve consensus by filtering information out of a noisy signal investigated by, amongst others, Poulakakis *et al*.^32^ and Fitch and Leonard^33^. Controllability can also be used as a measure to select nodes^34^.

The present study concentrates on the role of the FLE of the Laplacian to distribute tracking of the external driver across all the nodes so as to obtain the fastest convergence possible towards that driver. The tracking effort is considered to be globally limited, that is, only a fixed amount of attention can be paid to an external driver across the whole network (this constrains 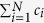). Under this hypothesis, the best distribution of effort across the nodes is sought to drive the network towards rapid consensus without allowing additional resources to be required. In contrast, the scenario in which all the nodes apply the same amount of effort would not take advantage of the group. A natural environment analogy would be to identify the key individuals within a group that should focus their effort on keeping a lookout for predators, allowing others to focus most of their effort on foraging. By exploiting the connections in the group it is possible to allocate duties efficiently amongst all of the group members.

We investigated and extended the concept of dynamical influence to cover not only which nodes should be in charge to lead the dynamics, but also how much effort they should invest in doing so. The model in Eq. (1) is used to identify the gain matrix *C* that guarantees the nodes achieve consensus about the external driver, whilst doing so in the shortest time possible.

A change of coordinates (see Methods) can be applied to Eq. (2) to isolate the matrix *L* + *C* as


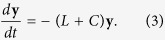


This model is used to find the diagonal matrix *C* that makes the dynamical system exponentially stable, with maximal rate and fixed effort. That is, all node states converge towards the same value with time and do so in the fastest way possible given a limited amount of effort. In contrast to the empirical approach taken by Klemm *et al*.^31^, here we consider an analytic approach providing mathematical proof of fast convergence. We show that the problem posed is solved by selecting the diagonal entries of *C* proportional to the entries of the FLE of *L*. Shang and Bouffanais^35^ used an analytic approach as well, concentrating their work on the number of interacting neighbours in a consensus problem with no external inputs. Instead we consider an external input, similar to the work by Shi *et al*.^36^. However, unlike Shi *et al*. this work looks beyond node selection towards the share of attention paid to the external input. The FLE of *L* is hence fed back into the system dynamics as a diagonal matrix to weight the control inputs towards tracking a uniformly fed external signal. As an eigenvector can be arbitrarily scaled the entries of the *C* matrix are scaled so that their sum is unitary. This allows comparison of the distribution with any other distribution summing to the same total, reflecting at the same time the global limitation considered.

## Results

The first result presented here guarantees that the nodes paying attention to the external signal are always able to drive the network to consensus. From a mathematical point of view this means proving that the eigenvalues of the matrix −(*L* + *C*) in Eq. (3) all have negative real part. A matrix with such characteristic is also known as a “Hurwitz matrix”. The matrix −*L*, on its own, is not Hurwitz due to the presence of the zero eigenvalue. By adding weights to its diagonal entries some nodes are effectively put in charge of tracking the external driver; hence, influencing the other nodes that observe them. It is then logical that the nodes in charge should be observed by all the others, or more generally are observed by some other nodes, which are in turn observed by the rest of the network. Nodes presenting this characteristic are called “globally reachable” or “globally observed”. Putting these nodes in charge, that is, assigning positive diagonal elements of *C* corresponding to the indices of globally reachable nodes, makes −(*L* + *C*) Hurwitz and guarantees the network will converge towards the desired state. This characteristic is illustrated through two examples in the Supplementary Information S3.

Choosing the elements of *C* proportional to the FLE of *L* makes the system Hurwitz since the elements of the FLE are positive in the indices corresponding to the globally reachable nodes. We note that as long as even just a single globally reachable node is provided with tracking capabilities the network will achieve consensus. Thus, any vector, having at least one positive component, where the same-indexed FLE entry is positive, would then produce a Hurwitz system matrix and a convergent system. The last statement is summarised and proven in Theorem S1 in the Supplementary Information S2.

It is now possible to show that distributing signal pursuing abilities so that they are proportional to the FLE entries for each node also provides near maximal consensus speed (see Methods for details). The speed of consensus is governed by the eigenvalue of −(*L* + *C*) that is the smallest in magnitude. The larger the magnitude of this eigenvalue, the greater the consensus speed. Our choice of *C* is particularly effective when the strength of interactions amongst the nodes are dominant with respect to the strength of the external driver. This is captured by a difference in magnitude between the Laplacian matrix *L* representing inter-node interactions and the matrix *C* weighting the external driver, i.e., for 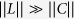.

To show this, consider again the system in Eq. (3) with an arbitrary diagonal matrix *D* with small nonnegative entries such that 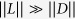:


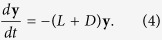


Let the matrix *M* be defined by adding a constant positive value to the diagonal entries of −*L*, such that *M* = −*L* + *kI* with *k* a real, positive scalar and *I* the identity matrix of appropriate dimension. As detailed in the Methods, the eigenvalues of *M* are equal to *k* added to each of the eigenvalues of −*L*, but the two matrices keep the same eigenvectors. For *k* large enough the matrix *D* that maximises the consensus speed also minimises the spectral radius *ρ*(*M* − *D*), which is the largest eigenvalue in magnitude of *M* − *D*. For 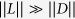 we get^37^


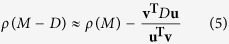


where **u** and **v** are the right and left eigenvectors of *M* relative to the spectral radius of *M*, respectively. Since the eigenvectors of *M* are the same as those of −*L* and the spectral radius of *M* corresponds to the zero eigenvalue of −*L*, then **u** = **1**, which is the vector of all ones, and **v** is the FLE of *L*. Thus, *D***u** = *D***1** is the vector of the diagonal elements of *D* and **u**^**T**^**v** is the *L*_1_ norm of **v**.

Now suppose the vector of the diagonal elements of *D* is restricted such that its *L*_1_ norm is equal to a generic positive scalar *α*. Taking *D* = *C* = diag{**v**}, with the FLE **v** scaled such that its *L*_1_ norm is *α*, all the eigenvalues of −(*L* + *C*) will be negative, and the dynamical system described by Eq. (4) will converge as per Theorem S1. Moreover, since *D***u** = *C***1** = **v** is in the direction of the gradient of the spectral radius of *M*, −**v**^*T*^, the choice of *D* = *C* provides a minimisation of the approximation (5) of the spectral radius of *M* − *D* and thus a near maximisation of the consensus speed of the system described by (4). The smallest eigenvalue in magnitude of −(*L* + *C*) is


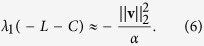


The magnitude of the smallest eigenvalue of −(*L* + *D*) cannot, in general, be maximised for *D* = *C* = diag{**v**} if the relative magnitude of *C* is large with respect to the magnitude of *L*. The FLE represents a (near) optimal choice when its magnitude is considerably smaller than the magnitude of the Laplacian such that Eq. (5) is very good approximation.

Equation (6) is obtained under the hypothesis that the diagonal vector of *D* has fixed *L*_1_ norm. If instead a fixed Frobenius matrix norm for the matrix *D* is assumed, a similar argument can be used to show that *D* = *C* = diag(**v**) maximises the spectral radius^38^ of −(*L* + *C*). This is shown in the Supplementary Information S5. Finally, if the fixed trace of the diagonal matrix *D*, as opposed to fixed norm, is considered, the optimal solution can be found independent of the magnitude of *D*, as described in the Supplementary Information S10. This is not done here as the fixed trace optimisation includes the possibility of negative weights, which would not be compliant with the present framework.

**Figure 1:**
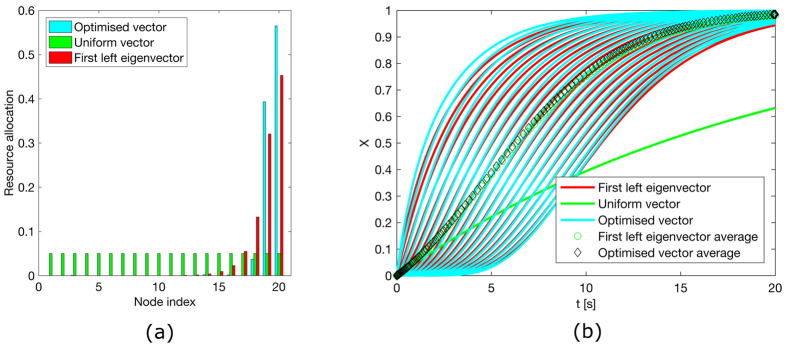
Consensus in a lattice and performance comparison. (**a**) Comparison among the values of the diagonal entries of matrix *D* for the matrix composed of the first left eigenvector, a vector obtained through numerical optimisation and one obtained by distributing evenly the tracking characteristics. All these vectors are scaled to have a unitary *L*_1_ norm. (**b**) Time evolution of the first order system driven to consensus about *x* = 1 by the diagonal perturbation of the Laplacian.

**Figure 2:**
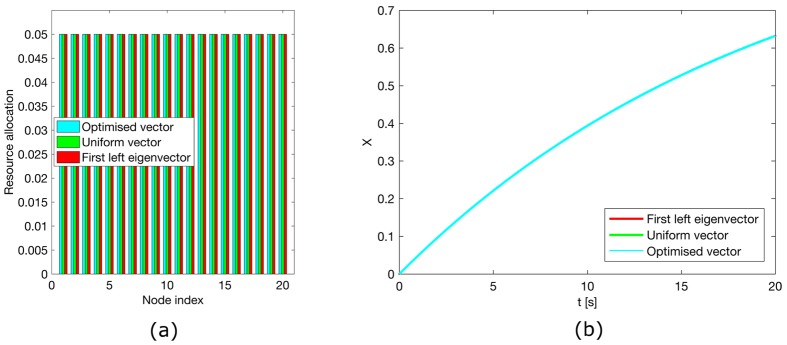
Consensus in a ring and performance comparison. (**a**) Comparison among the values of the diagonal entries of matrix *D* for the matrix composed of the first left eigenvector, a vector obtained through numerical optimisation and one obtained by distributing evenly the tracking characteristics. All these vectors are scaled to have a unitary *L*_1_ norm. (**b**) Time evolution of the first order system driven to consensus about *x* = 1 by the diagonal perturbation of the Laplacian. As the three distributions coincide the plots overlap.

**Figure 3:**
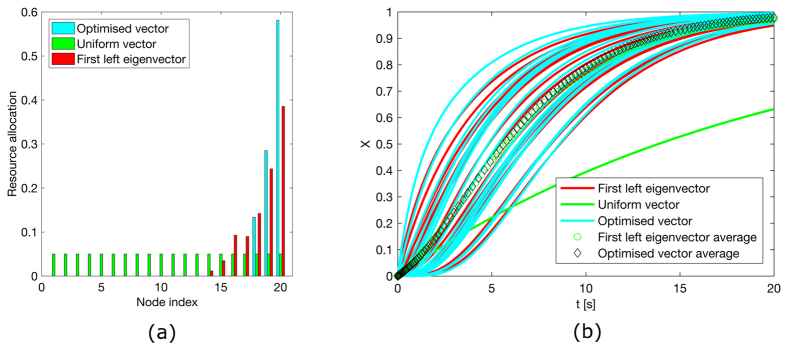
Consensus in a Small World network and performance comparison. (**a**) Comparison among the values of the diagonal entries of matrix *D* for the matrix composed of the first left eigenvector, a vector obtained through numerical optimisation and one obtained by distributing evenly the tracking characteristics. All these vectors are scaled to have a unitary *L*_1_ norm. (**b**) Time evolution of the first order system driven to consensus about *x* = 1 by the diagonal perturbation of the Laplacian.

**Figure 4:**
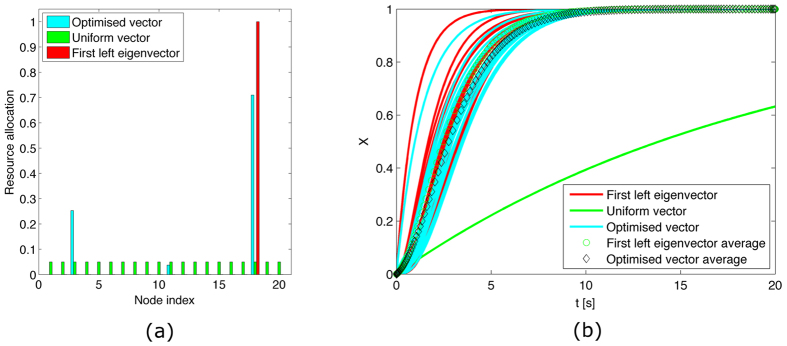
Consensus in a random network and performance comparison. (**a**) Comparison among the values of the diagonal entries of matrix *D* for the matrix composed of the first left eigenvector, a vector obtained through numerical optimisation and one obtained by distributing evenly the tracking characteristics. All these vectors are scaled to have a unitary *L*_1_ norm. (**b**) Time evolution of the first order system driven to consensus about *x* = 1 by the diagonal perturbation of the Laplacian.

Figures 1, 2, 3, 4 illustrate the theoretically proven effectiveness of the FLE **v** as a means to distribute tracking resources. The illustrations are obtained by numerically integrating the network dynamical system. The evolution of the system is shown for three distributions of the tracking resources assigned through the matrix *D*. The networks used are a non-symmetric lattice, a periodic lattice (ring), a random network, and a small world network. These are further illustrated in Methods.

In the figures, the distribution of resources proportional to the FLE is compared to a distribution obtained numerically, through a routine that searches for a distribution maximising the magnitude of the first eigenvalue. A third, uniform distribution of the tracking resources is also compared.

In the case of a ring graph, the FLE, the numerically optimised distribution, and the uniform distribution coincide. Indeed, for normal graphs like the ring the optimal resource allocation is a uniform one, regardless of the total amount of resources. For all the other cases the numerical optimal and the FLE distribution are biased towards nodes being observed more than observing others, hence their capability to efficiently influence the dynamics. In all these cases the convergence speed provided by a non-uniform distribution of the resources clearly surpasses that of the uniform distribution. In particular, the random network case presents only one globally reachable node. This is awarded all the resources by the FLE although the numerical optimiser finds a different resource allocation favouring two non-globally reachable nodes. However, both strategies quickly drive the network to converge. As shown in Table 1, the FLE achieves a consensus speed, measured by the smallest eigenvector in magnitude of −(*L* + *D*), comparable to the numerically optimised solution and significantly better then the uniform distribution.

**Figure 5:**
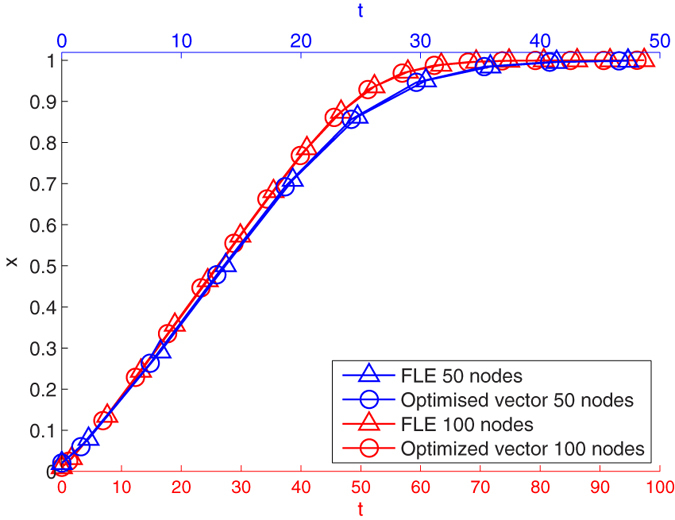
Time history of the average node state for the first order system driven to consensus about *x* = 1 by the diagonal perturbation of the Laplacian. 50 and 100 node systems are considered. Scaling the integration time with the nodes returns very similar time histories, regardless the size of the systems. For each system both the dynamics given by the FLE and by a numerically optimised resource distribution are plotted. The blue curves refer to the 50 node system and are plotted against the blue horizontal axis at the top. The red curves are plotted against the red horizontal axis at the bottom.

The resource distribution based on the FLE offers performance independent of the number of nodes, when compared to a numerically optimised distribution. Figure 5 illustrates this. In the figure, the same kind of lattice network used in Fig. 1 is considered, and extended to 50 and 100 nodes. The average state, that is the average over the node states at each time step, is plotted against time. As for Fig. 1, this is done for the FLE and the optimised resource distribution. Scaling the time with the nodes shows how the behaviour is unaffected by the size of the network. In particular the difference with respect to the numerically optimised result is small enough to observe that the plots are superimposed almost everywhere.

### Extension to second order dynamics

The prior results are extended to more closely replicate real world dynamics where the network nodes are agents moving in a three-dimensional space. The agents attempt to align their velocity with the external driver, whilst keeping relative spacing. Consensus happens on two variables for each agent: one is the velocity, the other is the relative position, which vary with the trajectory taken. It is then a second order system that describes the dynamics of the generic *i*th agent:


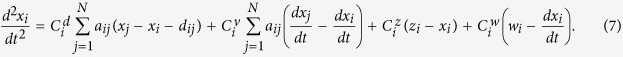


The symbols are the same as those used for the first order system and the coefficients *c*_*i*_ of the first order system are substituted by the constants 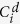, 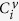, 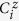, 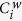 that weigh the relative importance of the terms of the equation. The parameter *z*_*i*_ represents the goal for the actual position *x*_*i*_ and *d*_*ij*_ is a desired gap between the positions of any two agents *i* and *j* connected in the network. For cooperative agents, *d*_*ij*_ should equal −*d*_*ji*_, although this is not strictly necessary. The second order system is evaluated using a numerical simulation of the dynamics, physical intuition, and an analysis of the spectral properties of the system matrix.

Equation (12), the vector form of Eq. (7), describes the evolution of the network of agents achieving consensus about relative position and velocity. For the extension to the second order system, only the regular lattice case is considered. For this case, the sharing of the finite amount of resources should include tracking an external driver, a desired velocity but not a desired position (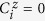 for all *i* = 1, 2….*n*, see Methods). Moreover, the sharing of resources should also include the social terms producing consensus about relative distances and velocities, that is 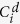 and 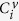. Only one sharing ratio amongst 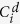 and 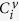 is considered, with the relative importance being quantified by the parameter *κ*, as explained in the Methods. More cases are considered in the Supplementary Information S9.

**Figure 6:**
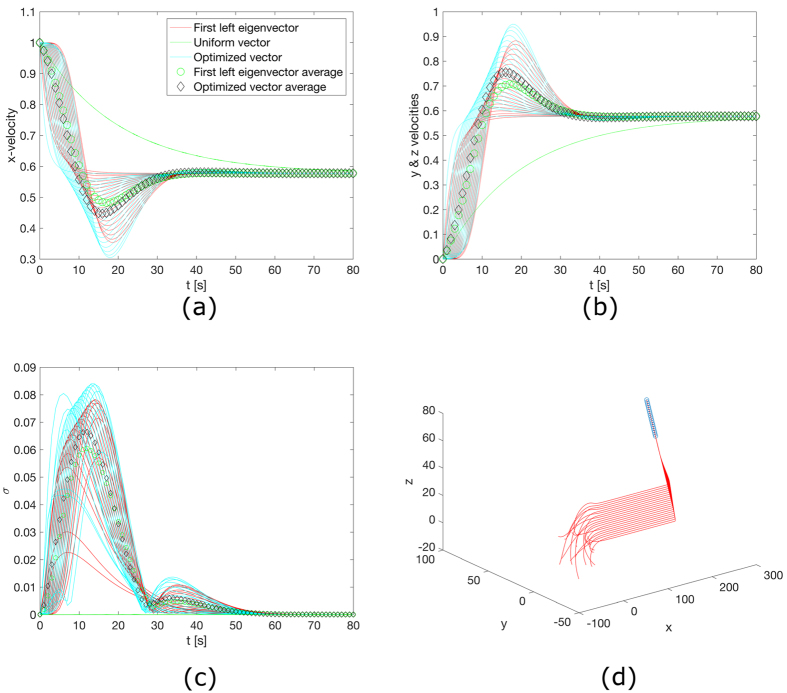
Consensus in a lattice network, for a second order system for *κ* = 0.1 (see Methods). (**a**) Consensus about *x* velocity. (**b**) Consensus about *y* or *z* velocity. (**c**) Standard deviations of the agent relative distances over time showing consensus about common reciprocal separation. (**d**) Trajectories in a physical space of the agents from the initial random state (initial position in a sphere of radius *N*/2 and initial velocities in the unit sphere) to consensus about a common direction and relative distances. Step change in target velocity produces the bend and the alignment to the direction 
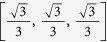
 at time 0. The low value of the parameter *κ* guarantees low overshoot.

The system behaviour is reported in Fig. 6, where the consensus about the common desired velocity along the three axes, the standard deviation about the targeted inter-agent distance for each agent, and a representation of the motion in three-dimensional space are shown. The rise time and the settling time were considered as well. These are reported in Table 2. Note that the change in *y* and *z* velocities are the same and are hence combined together in the plot and the Table.

The state space description provides a unified matrix for the relative positions and velocities. This gives a measure of the system convergence speed, as for the first order system. Unlike the first order system, the second order matrix keeps the zero eigenvalue as a consequence of the indefinite agent positions, which are set only in terms of relative distances. Consensus speed is again evaluated through the smallest in magnitude, non-zero eigenvalue of matrix *S* (See Eq. S14 in the Supplementary Information). This is reported in Table 3 while the effects on stability properties and the existence of the zero eigenvalue are covered in the Supplementary Information S6–S8.

## Discussion

Assigning tracking resources proportionally to the entries of the FLE enhances the pursuit of an external signal and nearly maximises the speed of consensus about it. In proposing and investigating this resource allocation strategy the present work reveals features of this method and makes a comparison with classical optimization techniques. The comparison can be made in terms of time, computational effort and accuracy.

While the effort in computing the FLE is a function of the number of nodes and their connections, the efficiency of the numerical optimization process relies on the initial guess and the algorithm used. This is true in case of centralised computation and even more relevant when distributed strategies are adopted. Moreover, possible poor conditioning of the numerical optimisation problem should be taken into account. A resource assignment proportional to the FLE provides performance very close to the numerical optimal, with the advantage of not requiring an initial guess and achieving a guaranteed, near optimal consensus speed.

If the FLE were to be used in the context of distributed engineering systems (e.g. mobile robotic agents), then its calculation could be achieved in a distributed way. The distributed, on-line calculation of the FLE is beyond the scope of this paper. There are a number of methods proposed in the literature, which could be applied to computing the FLE on-line. These methods are well suited for moderately sized distributed networks; however, they are not yet practical for implementation in large distributed systems. For example, the power iteration method has served as the basis for several algorithms^39^,^40^,^41^,^42^,^43^. These algorithms, in most implementations, make the network nodes achieve a consensus about the FLE and the Fiedler value of the Laplacian matrix. They require the graph connectivity, as in our case, and a network topology changing more slowly than the convergence time. In particular Di Lorenzo and Barbarossa^41^ include the possibility of link failures through a stochastic approach. The algorithm proposed by Qu *et al*.^43^ offers good performance against the presence of possible communication delays. Finally, Bertrand and Moonen^40^ provide a measure of the complexity of their algorithm with a number of floating point operations per node increasing linearly with the size of the node’s neighbourhood. These algorithms, however, do not scale well in performance when the number of nodes increase beyond a few tens. The time needed to converge makes the distributed calculation not suitable for the time scales of many real world applications. The algorithm proposed by Salahi *et al*.^42^ exploits the wave equation to extract the components of the FLE through a Fast Fourier Transform and this way provides a very fast algorithm. However the methods can easily be affected by noise, which cannot be disregarded in the context of real engineering systems. In this respect Di Lorenzo and Barbarossa^41^ argue that the stochastic power iteration in their algorithm asymptotically reduces the noise variance, i.e. the detrimental effects of noise. The noise problem is a general one as this would affect all computations taking place over the network, including any optimization routine used in place of the FLE.

The present study work considers nodes receiving an undisturbed external signal and communicating with their neighbours without any interference. As such, the problem considered does not take into account the effect of noise in communication on the dynamics. However, if noise were considered, the global limited resource framework is even more compelling. Suppose that each agent can only measure a noisy version of the true signal. Suppose further that the amount of noise agents receive is inversely proportional to their investment, or at least monotonically decreasing for an increasing investment, which could relate to power usage in the sensors or the sensor cost itself. In this case, agents with a higher investment (and higher cost) receive a better signal, and so can allocate a higher gain to tracking that signal. Then, to maximise the efficiency of the group, it is advisable to keep the total investment, i.e., the sum of the investments of each agent, as low as possible, or, alternatively (as is done here), achieve the maximum possible performance for a given level of total investment. In this scenario, having tracking gains sensibly smaller than the social term, where the FLE allocates them optimally, makes sense in the case of a very poor/noisy signal relative to the inter-agent communication. Collective migratory behaviours influenced by noise have been analysed using graph theory^20^, while the complementary aspect of minimizing noise effects on the steady state variance of the group through leader selection has recently been tackled^33^. However, neither work considers the convergence speed as we do in the present paper.

The second order system presented is a more compelling case for the problem of resource allocation as it presents, for the same agents, more dynamics to control and hence sets a limit on the amount of resources each agent can provide. The second order system, not surprisingly, shows how the optimization of the consensus speed about an externally fed velocity contrasts with the achievement of consensus about inter agent distances. Consequently, concentrating more social interaction towards the speed consensus, i.e. reducing *κ*, pushes the system behaviour towards that of the first order system. *κ* = 0 would eliminate the oscillatory behaviour visible in Fig. 6a,b, but would also imply no control of inter-agent distances. The increase of the inter-agent distance social term makes the system more rigid as the lower left partition of the matrix in Eq. (16) prevails over the lower right partition. Considering the simplified case of coupled damped oscillators, this would make the rigidity terms progressively larger with respect to the damping term, hence the faster, more oscillatory behaviour.

A general consideration emerges by comparing the performance of different graphs. Unbalanced graphs can have significantly improved performance in terms of convergence speed compared to balanced ones, which, aside from the biological motivation, demonstrates why directed, and unbalanced directed, graphs should be favoured for fast convergence. A noteworthy consequence is that the optimal diagonal matrix with fixed *L*_1_ norm of a complete graph on *N* nodes will have entries 1/*N*, which is also the magnitude of the dominant eigenvalue of the resulting system. This leads to the associated dynamical system becoming slower in achieving the goal as the number of nodes increases. Conversely, reducing the number of connections and targeting just a few leading nodes can produce faster dynamics. The class of balanced graphs is not restricted to the circulant graphs (an example of which was also detailed in the Results section), nor to highly symmetric graphs. Other, less intuitive, balanced graphs produce the same slow convergence observed in the ring case whereas the uniform distribution indicated by the FLE remains the one maximising the convergence speed.

In limiting the amount of resources across the group, we take the perspective that it is costly for an agent to obtain information directly from the environment and considerably less costly to rely on social cues. This is consistent with studies in the literature on collective animal behaviour. For example, Guttal and Couzin^44^ used this perspective to study collective migration and the evolution of effort across the collective in obtaining information on a migratory direction; they assumed that to obtain the migratory direction, individuals pay for example in energy expenditure and reduced predator vigilance. We have shown that under the constraint of such a cost, fastest consensus is achieved with a non-uniform distribution of reactivity to the external signal. In the case of the second order, spatial system, for instance, a uniform distribution of signal pursuit duties over-constrains the swarm’s ability to manoeuvre and results in slower responses to threats or manoeuvres towards a target. Instead, it is sensible to exploit the group dynamics so that the more reactive agents drive swarm manoeuvres. Such a targeted distribution of resources releases some agents from signal pursuit duties leaving them free to allocate resources to other tasks, such as predator vigilance or foraging. This consequently relaxes the constraints on the system while it results in faster responses to threats or manoeuvres towards a target.

In the present study, only time invariant networks have been considered. When looking at real systems, in particular those comprising mobile agents, these would continuously rewire their connections based on “who sees whom”. The role of the changing network topology on system behaviour has been the subject of recent research; see the review by Gross and Blasius^45^. However, deriving analytical results on performance of a system determined by a changing network topology remains an open, challenging problem. In the present work, we account for a changing network topology by assuming a time scale separation in which the evolution of the network topology is slower than the time required to compute and update the resource allocation. In particular, we assume that the near-optimally of the FLE allocation remains during the period of time between allocation updates. This may be particularly true during collective manoeuvres. Consider, a fish school following a faint chemical plume or a warm subsea stream. When the school manoeuvres, the graph of interactions changes continuously, however, it is conceivable that from the time the manoeuvre is initiated to the time the school has engaged with it, the graph of interactions has not changed significantly. That is the graph that matters may be the one that agents consider when weighting their attention towards the signal. In this respect a parallel with the biological world can be justified in some cases.

The spectral approach proposed here lends itself well to engineering exploitation, although the challenges arising in real time applications must be addressed. On the other hand, this approach finds inspiration and mirroring in the natural world. While it is unlikely that biological flocks rationally choose the amount of attention to pay to their neighbours, it is conceivable that evolution has provided good solutions. This has been verified for the number of topologically interacting individuals in starling flocks; analysis suggests that evolution may have produced a number maximising the robustness of consensus to noise^46^. In the same way, it would be interesting to compare the evolutionary solution to the limited resource allocation in the pursuit problem.

## Methods

### General Methods and First Order Dynamics

Directed graphs (digraphs) with general typology, on *N* nodes, are considered. The network dynamics are expressed through the classical Laplacian matrix of the digraphs. With no external input the system would evolve reducing the differences in the states of the connected nodes and, eventually, achieving a final state, common to all the nodes. We consider the case in which a constant external input signal *w* is received by each node *i* with different magnitudes determined by the gains *c*_*i*_ ≥ 0, *i* = 1, …, *N*. This is modelled by Eq. (1). For the sake of clarity, in the first order system, a one-dimensional state is considered although the results can be extended to an *n*-dimensional state without loss of validity.

If a parallel was made with biological or engineering swarms, the allocation of a fixed amount of resources across the group is equivalent to electing individuals for signal pursuing and leaving others to look after other tasks. Another fundamental assumption in this analysis is that 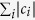, known as the *L*_1_ norm, is a fixed, given value. This implies that the signal pursuing duty is limited at the group level rather than the individual level. Since *c*_*i*_ ≥ 0, the *L*_1_ norm is equivalent to 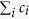.

The Laplacian matrix is defined as


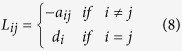


where *d*_*i*_ is the outdegree of node *i*, that is the number of edges that have origin in *i*. Eq. (2) is transformed to Eq. (3) through the change of coordinates


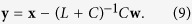


This eliminates the term *C***w**, or equivalently, sets the origin as the input.

The results on the consensus speed are obtained by investigating the eigenvector corresponding to the spectral radius, that is the largest eigenvalue in magnitude, of a matrix *M* − *D*, where *M* = −*L* + *kI, I* is the identity matrix, and *D* is a diagonal matrix with nonnegative diagonal elements. The spectrum of any matrix is shifted by the constant quantity *k* if a uniform, diagonal perturbation *kI* is added. Hence the spectrum of *M* is shifted by *k* with respect to the spectrum of −*L*. The same applies to the matrices −*L* − *D* and *M* − *D*. The eignevectors, however, are not affected by the shifting.

Assume that the matrix −(*L* + *D*) is Hurwitz, that is all its eigenvalues have negative real part, and that the scalar *k* is real, positive and larger than the largest eigenvalue in magnitude of −(*L* + *D*). This makes *M*−*D* = −*L* − *D* + *kI* a strictly positive matrix with the smallest eigenvalue in magnitude of −(*L* + *D*) corresponding to the spectral radius of −*L* − *D* + *kI*, namely *ρ*(*M* − *D*). Hence the *D* that minimizes *ρ*(*M* − *D*) corresponds to the *D* that maximizes the smallest eigenvalue in magnitude of −(*L* + *D*) and thus maximises consensus speed. In particular, given a generic *N* × *N* matrix *B* its spectral radius is an always increasing function of each entry of *B*, and in particular the expression


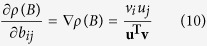


holds, where **u** and **v** are the first right and left eigenvector respectively of *B* associated to its spectral radius^37^. The entries of both **u** and **v** are nonnegative.

Using Eq. (10) the linearisation of the spectral radius of *M* − *D* can be expressed as a function of the matrix entries as in Eq. (5). The linearisation is a good approximation of the spectral radius of *M* − *D* when 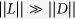.

Now let *D* = *C* = diag{**v**}, where **v** is the FLE of *L* with *L*_1_ norm equal to positive scalar *α*. Let **u** = *u***1**, *u* = *β*/*N*, then **u** is the first right eigenvector of *L* with *L*_1_ norm equal to positive scalar *β*. Let 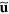 and 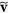 be the same vectors scaled such that each has unitary *L*_1_ norm, i.e.,


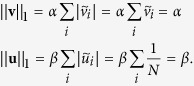


Further assume that the system has high coupling gains, that is 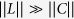 for some norm ||·||. Then,


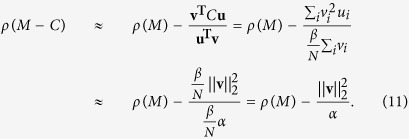


In particular, as the rightmost eigenvalue of −*L* is zero, Eq. (11) implies Eq. (6).

### Second order system

Eq. (7) can be written in vector form to evaluate the consensus speed of the second order system. Assume that agents do not have a preference, or a driving input for their positions in absolute terms, that is 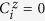, for all *i* = 1, 2….*N*. Then, Eq. (7) can be written in vector form as


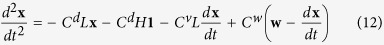


where, *C*^*d*^, *C*^*v*^ and *C*^*w*^ are diagonal matrices, **w** = *w***1** as before, with *w* a desired common speed. *H* is a matrix of the edge-weighted desired distances between any two connected agents. For illustrative purposes, *H* can be defined so that the physical distance between two agents tracks the topological distance, that is


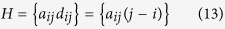


where *a*_*ij*_ is the entry of the adjacency matrix, as previously stated. Note that *H* can have negative entries as this is the distance with sign between the indices of two connected nodes in the graphs (e.g. if there is an oriented edge from node 4 to node 1, then *H*(4, 1) = −3). Matrices *C*^*d*^, *C*^*v*^ and *C*^*w*^ are chosen so as to comply with the bound on the total amount of resources allocated. Moreover, agents are forced to seek velocity consensus by ensuring that not all the resources are allocated to the inter-agent distance keeping task only. This is expressed by the relation


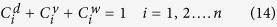


with the condition


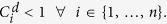


This second order model can be translated into the state space using the variables **r** and **s** such that **x** = **r** and *d***x**/*dt* = **s**. The resulting state space form is


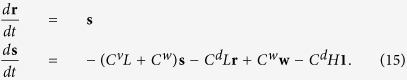


Using matrix notation, Eq. (15) becomes


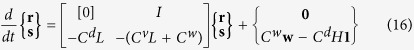


where *I* is the identity matrix of appropriate dimension, while *C*^*d*^, *C*^*v*^ and *C*^*w*^ are diagonal matrices weighting the social and external contributions. The system matrix in Eq. (16) has one zero eigenvalue as a consequence of the fact that just the relative distances between the agents are fixed but not the positions in absolute terms. This is proved in the Supplementary Information S6. The stability analysis was performed numerically for the second order system and restricted to the case of an asymmetric lattice. The analysis includes an assessment of the influence of the resource sharing between the social terms of position and velocity and is reported within the Supplementary Information S7 and S8.

For the rise time and the settling time considered in Table 2, the accuracy is ±0.05 *s*. The inter-agent distance performance is evaluated through the settling time for the agents’ mean deviations from their intended inter-agent distances. The inter-agent distances are considered settled when this value drops within 5% of the peak value achieved during the simulation. As the change in velocity requested in the motion along *y* and *z* axes is the same, their dynamical response is identical and are hence combined together in the plot and the Tables.

### Graphs

**Figure 7:**
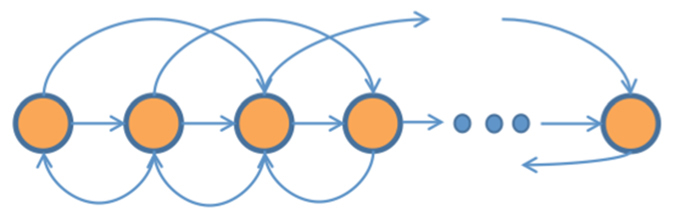
Sketch of the lattice network. Each node in the inner section has 3 outgoing and 3 incoming edges. Nodes at the left hand side have more outgoing than incoming edges, while nodes at the right hand side end have more incoming than outgoing edges. Note that an outgoing edge implies that the node where the edge is originated “observes” the node where the edge ends.

The graphs considered here are directed, “sensing graphs”: an edge *ij* implies that node *i* senses, or gathers information on, the state of node *j*. Whilst the analytic development is independent of the graph and just based on the hypothesis of connectedness, the numerical simulations are run using four graphs of interactions. The first one, on which the second order dynamics simulations are based too, is an asymmetric lattice. This can represent individuals all looking in the same directions (say forward), and focussing their attention on a restricted number of neighbours found in their field of view. This finds justification in the preferential direction of view found in animal groups and in the attention being concentrated on a restricted group of individuals in particular areas within their sensing range^46^. Here it was chosen to give preference to the forward sensing more than the backward sensing within a maximum sensing range of two subsequent individuals. A lattice is a plausible, though somewhat rigid, representation of the sensing links in groups where sensing range is limited. The sketch in Fig. 7 represents the linking scheme.

The ring graph and the small world graph are derived from the asymmetric lattice by either transforming the adjacency matrix of the asymmetric lattice into a circular one or rewiring, with probability 0.1, the links of the asymmetric lattice. The random graph is created by setting links between any two agents with a probability arbitrarily set to 0.15.

### Numerical Simulations

The dynamics are integrated using the Dormand-Prince method^47^. The numerically optimised resource distribution is obtained through a constrained nonlinear optimization, using an interior point method. The initial guess for the optimization is the first left eigenvector, so that the optimizer cannot perform any worse than the result obtained through the FLE. In the second order system, to study the sensitivity of the outcome to a simple parametrization of the 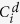 and 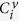 values, these are defined as


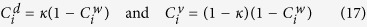


using the free parameter *κ* ∈ [0, 1], which limits to unity the total amount of resource allocated to each single agent.

In test cases the system is allowed to relax, starting from random initial positions distributed on a sphere of radius *N*/2, with velocities on the unit sphere. The agents achieve uniform distances from their neighbours and uniform velocity along the positive x-axis, both set to be unitary in magnitude. The swarm is then subject to a step-like input in speed along the vector 
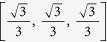
 at time 0. The simulations are run for 200 s prior to time 0 during which the system evolves from random initial conditions to achieving a uniform velocity distribution along the x-axis and uniform spacing. Then the stimulus is fed to the system and the simulations are run for a further 80 s.

The rise time is defined as the time elapsed for the average group velocity to match the target value, regardless of the overshoot. The settling time is defined as the time to stabilise the average of either the group velocity or the inter-agent distance, both within 5% of their target value.

## Additional Information

**How to cite this article**: Punzo, G. *et al*. Using Network Dynamical Influence to Drive Consensus. *Sci. Rep.* **6**, 26318; doi: 10.1038/srep26318 (2016).

## Supplementary Material

Supplementary Information

## Figures and Tables

**Table 1:** First eigenvalues of the system matrix (*L* + *D*).

	FLE	Uniform	Optimised
Lattice	0.2994	0.05	0.3256
Ring	0.05	0.05	0.05
Small World	0.2416	0.05	0.2466
Random	0.6765	0.05	0.7098

*L* is the network graph Laplacian, *D* is a diagonal matrix whose nonzero entries are either the first left eigenvector (FLE), a uniform vector or a vector numerically optimised to maximise the correspondent eigenvalue. All these have unitary *L*_1_ norm.

**Table 2:** Rise time and settling time for the system in Eq. 7 subject to a step input along all three axes.

Rise/settling times [s]	FLE	Uniform	Optimised
*κ* = 0.1			
x velocity	9.55/27.65	−/53.70	9.55/27.55
y and z velocities	10.75/29.55	−/59.90	9.55/28.85
distance	42.40	>80	42.10
*κ* = 0.5			
x-velocity	7.25/44.75	−/53.70	6.95/45.35
y and z velocities	7.25/45.65	−/59.90	6.95/45.95
distance	48.60	>80	48.80
*κ* = 0.8			
x-velocity	6.45/>80	−/53.70	6.05/>80
y and z velocities	6.45/>80	−/59.70	6.05/>80
distance	53.75	>80	63.15

The accuracy is ±0.05 *s*. The inter-agent distance performance is evaluated through the time taken by the average of all the mean deviations of the agents from their intended inter-agent distances to stabilise within 5% of its peak value. In the “uniform” case, the relative distance does not reach the 5% of its maximum value because it is nearly constant as a result of all the agents being provided with the same input. At *κ* = 0.8 the rise time is short, but the system takes a long time to settle. In the numerical tests this was not achieved within the 80 seconds over which the system dynamics was simulated.

**Table 3:** Smallest (in magnitude) eigenvalues of the system matrix for the second order system in its state space form.

	FLE	Uniform	Optimised	*κ* value
Lattice	0.0931	0.05	0.1142 ± 0.0007*i*	*κ* = 0.1
	0.1110 ± 4525*i*	0.05	0.1104 ± 0.4520*i*	*κ* = 0.5
	0.0460 ± 5877*i*	0.05	0.0461 ± 0.5857*i*	*κ* = 0.8
Ring	0.05	0.05	0.05	*κ* = 0.1
Small World	0.1006	0.05	0.1140 ± 0.0004*i*	
Random	0.1136 ± 0.0002*i*	0.05	0.1137	

As for the first order system the allocation of the tracking resources is considered according to the first left eigenvector (FLE), a numerical optimised distribution and a uniform one. All these have unitary *L*_1_ norm.
