# Supplementary material for: Using Network Dynamical Influence to Drive Consensus
Source: Sci Rep. 2016 May 23;6:26318. doi: 10.1038/srep26318 (PMC4876330; doi:10.1038/srep26318)
Supplement: Supplementary Information [file srep26318-s1.pdf]

SUPPLEMENTARY MATERIALS
Using Network Dynamical Influence to Drive Consensus

Giuliano Punzo[1,3*], George F. Young[2], Malcolm Macdonald[1], Naomi E. Leonard[2]
1 Department of Mechanical and Aerospace Engineering, Advanced Space Concept Laboratory, University of Strathclyde, Glasgow, Scotland, UK
2 Department of Mechanical and Aerospace Engineering, Dynamical Control System Laboratory, Princeton University, Princeton, NJ, USA
3 Department of Civil and Structural Engineering, The University of Sheffield, Sheffield, UK
* E-mail: Corresponding giuliano.punzo@strath.ac.uk

S1 Equivalence between the Perron vector and the First Left Eigenvector

The Laplacian matrix L of a connected graph always has one zero eigenvalue. This eigenvalue can be linked to the spectral radius of a positive matrix obtained by adding a constant value to the diagonal entries of $-L$. This provides an extension of the Perron-Frobenius theorem to $-L$, where the spectral radius of the original formulation is replaced by the zero eigenvalue. Lemma S1 addresses this.

Lemma S1. *Let L be the Laplacian matrix of a connected graph. Then there exists a left eigenvector $\mathbf{v}$ corresponding to the zero eigenvalue such that every element of $\mathbf{v}$ is non-negative.*

Proof. Consider the matrix $M = kI - L$, where I is the identity matrix and $k \in \mathbb{R}$. Then the eigenvalues of M are equal to the eigenvalues of $-L$ plus k, and the eigenvectors of M are equal to those of L. By the Gershgorin theorem and the construction of L, all eigenvalues of L must lie inside discs centred on the positive real axis and passing through the origin. Furthermore, L has a zero eigenvalue (with right eigenvector $\mathbf{1}$). Therefore, if k is taken to be larger than twice the largest diagonal element of L, all eigenvalues of M will lie in the right half-plane, and the spectral radius of M will correspond to the zero eigenvalue of L. But under these conditions, every element of M is non-negative, and so by the Perron-Frobenius theorem, the desired left eigenvector $\mathbf{v}$ exists. □

S2 Hurwitz system matrix - Theorem S1

Through the first left eigenvector the most influential nodes can be identified and ranked. If an entry i of the FLE is larger than the entry j, this means that node i has more influence on the swarm dynamics than node j. It then suggests awarding the nodes corresponding to higher entries of the FLE more ability to purse a signal. By doing so, no pursuing capabilities are given to those nodes that are not observed by any other nodes, and hence cannot contribute to the system's convergence towards the goal. This is shown through Theorem S1

Theorem S1. *Let $L \in \mathbb{R}^{N\times N}$ be the Laplacian matrix of a connected graph as defined in Eq. (8) and let $\mathbf{v}$ be the FLE. The FLE is the left eigenvector of L corresponding to the zero eigenvalue, i.e., $\mathbf{v}^T L = 0$. Let the matrix $D \in \mathbb{R}^{N\times N}$ be a diagonal matrix whose entries are the elements of the nonnegative vector $\mathbf{d}$, i.e., $D = \operatorname{diag}\{\mathbf{d}\}$. Then $-L^* = -(L + D)$ is Hurwitz if and only if the scalar product $\langle \mathbf{d}, \mathbf{v}\rangle \neq 0$. In particular, if the matrix $C \in \mathbb{R}^{N\times N}$ is defined as $C = \operatorname{diag}\{\mathbf{v}\}$, then $-\tilde{L} = -L - C$ is Hurwitz.*

In order to prove Theorem S1, the following Lemmas are needed.

Lemma S2. *Let L be the Laplacian matrix of a connected digraph and let* $\mathbf{v}$ *be its first left eigenvector corresponding to the zero eigenvalue. Then* $\mathbf{v}$ *has a zero component v_i if and only if node i has only outgoing edges or for each edge $j - i$ entering node i with nonzero out-degree, $v_j = 0$ holds.*

The lemma implies that all nodes i that correspond to $v_i = 0$ are not globally reachable.

Proof. Consider the component i of the vector $\mathbf{v^T L}$

$$[\mathbf{v}^T L]_i = v_i L_{ii} + \sum_{j \neq i} v_j L_{ji} = 0. \tag{S1}$$

By hypothesis the $j = i$ term, that was taken out of the sum, is zero. From the Perron-Frobenius theorem, all elements of $\mathbf{v}$ are nonnegative (see Lemma S1), while the off-diagonal elements $L_{ji} (j \neq i)$ are all non-positive. Hence for the sum in Eq. (S1) to be zero, it must be that $v_j = 0$ for each j corresponding to an incoming edge $j - i$. Considering the same equation the reversed implication is evident. □

The lemma also implies that if a node is globally reachable, it must have $v_i \neq 0$. Indeed, looking at Eq. (S1) a node globally reachable must have at least one incoming edge L_{ji} corresponding to a $v_j \neq 0$. Hence the equation must be satisfied for some nonzero, and therefore positive, value of $v_i L_{ii}$. The only other possible case arises in a connected graph with a single globally reachable node. Calling this node i, it will be that $L_{ii} = 0$ as the node will have zero outdegree. As i is the only globally reachable node, $v_j = 0, \quad \forall j \neq i$. However, in this case v_i must be nonzero, as otherwise $\mathbf{v}$ would equal $\mathbf{0}$, which is not allowed as an eigenvector.

Lemma S3. *Let L be the Laplacian matrix of a connected digraph and let* $\mathbf{v}$ *be its first left eigenvector corresponding to the zero eigenvalue.* $\mathbf{v}$ *has a zero component $v_i = 0$ if and only if node i is not globally reachable.*

Before proving the lemma, it is the case to stress that the graph is required to be just connected and not necessarily strongly connected. In a connected graph there is at least one globally reachable node. Conversely, in a strongly connected graph, all nodes are globally reachable, that is, there is an oriented path connecting any pair of nodes. Moreover, the Laplacian matrix of a strongly connected graph is irreducible.

Proof. The first implication is a consequence of Lemma S2. More precisely, if $v_i = 0$ Eq. (S1) can be satisfied either for $L_{ji} = 0$ for all $j \neq i$ or for $v_j = 0$ for $L_{ji} \neq 0$. In the first case the node would not be reachable from any other node. In the second case, Lemma S2 can be recursively applied to each such node j and (eventually) every node that can reach node i through some path. This implies that if node i is globally reachable, then we must have $\mathbf{v} = \mathbf{0}$. However this is a contradiction. Hence, for $v_i = 0$, node i cannot be globally reachable.

For the reverse implication, in order to have non globally reachable nodes, by definition the graph must not be strongly connected. The Laplacian matrix L in this case is reducible and, through permutations, it is possible to arrange it in the form

$$L = \left[\begin{array}{c|c} \mathcal{L}_0 & B \\ \hline 0 & L_{GR} \end{array}\right] \tag{S2}$$

where $\mathcal{L}_0 \in \mathbb{R}^{l \times l}$ and $L_{GR} \in \mathbb{R}^{m \times m}$ with $N = l + m$ are square matrices whose rows refer respectively to non-globally reachable and globally reachable nodes. [0] is a matrix of consistent dimensions whose entries are all zeros and [B] is a matrix of consistent dimensions with no relevant characteristics. Note that $\mathcal{L}_0$ is not a Laplacian, as the row-sums of L must be zero and include also the entries of B. Conversely, L_{GR} still is a Laplacian, since its row sums equal zero (and it contains non-negative diagonal entries and non-positive off-diagonal entries). The eigenvalues of L are the union of the spectra of $\mathcal{L}_0$ and L_{GR}, and

because the graph is connected L has one single eigenvalue equal to zero that hence must belong to the spectrum of L_{GR} as this is a Laplacian itself. Consider a partition of the first left eigenvector accordingly, that is the first l and the second m entries as

$$\mathbf{v} = \left[\begin{array}{c:c} \mathbf{v}_{NGR} & \mathbf{v}_{GR} \end{array} \right]^T . \tag{S3}$$

As $\mathbf{v}_{NGR}^T \mathcal{L}_0 = 0$ must hold, and zero is not an eigenvalue for $\mathcal{L}_0$, this means that $\mathbf{v}_{NGR} = \mathbf{0}$. □

Lemma S4. *Let L be the Laplacian matrix of a connected digraph and let $\mathbf{v}$ be its first left eigenvector corresponding to the zero eigenvalue. If $\mathbf{v}$ has a zero component $v_i = 0$ then the diagonal element of the Laplacian matrix $L_{ii} \neq 0$.*

Proof. The lemma can be proved by contradiction by noting that if both $v_i = 0$ and $L_{ii} = 0$ then the node i would have no outgoing edges and all its incoming edges would start from a non globally reachable node. Hence the node i would belong to an isolated component and the graph would be disconnected. □

It is now possible to prove Theorem S1. First the theorem is proved for the case of the diagonal matrix $D \equiv C = \text{diag}\{\mathbf{v}\}$, that is, being composed of the FLE. After this, the proof is extended to the more general case of $D = \text{diag}\{\mathbf{d}\}$ with $\langle \mathbf{d}, \mathbf{v} \rangle \neq 0$.

Proof. First suppose L to be reducible and consider its partition as in Eq. (S2). The first l columns of L correspond to those nodes with index i for which $v_i = 0$. All incoming edges to those nodes must have their origin in nodes indexed by some j for which $v_j = 0$ too. So all the nonzero entries of the first l columns must be in the top left partition $\mathcal{L}_0$. As the zero eigenvalue is found in L_{GR}, $-\mathcal{L}_0$ is Hurwitz. It follows that the matrix $-\tilde{L} = -L - C$ is Hurwitz too because the zero entries of $\mathbf{v}$ leave $\mathcal{L}_0$ unchanged and the positive ones make the matrix $-L_{GR} - C_m$ Hurwitz, where C_m is the diagonal matrix made up of the last m components of $\mathbf{v}$. This follows as a simple consequence of the Gershgorin disk theorem. The case of L being irreducible, can be considered as a particular one where $l = 0$.
Consider now the more general case of $D = \text{diag}\{\mathbf{d}\}$ with $\langle \mathbf{d}, \mathbf{v} \rangle \neq 0$. As both $\mathbf{d}$, $\mathbf{v}$ are non negative, for their scalar product to be non zero it must be that $d_i, v_i \neq 0$ for at least one index i. If this is the case, considering the matrix L partitioned as in Eq. (S2), some nonzero elements of D would add to the lower partition L_{GR} making at least one row sum of the resulting matrix positive. Furthermore, again from the Gershgorin disk theorem, nonzero elements of $\mathbf{d}$ added to the top left partition $\mathcal{L}_0$ will leave it Hurwitz. The proof is completed by considering Theorem III in [Tau49], which is reported here for the sake of completeness with the notation used so far.

Theorem S2. *([Tau49])*
Let $L \in \mathbb{R}^{N \times N}$ such that $L_{ii} \geqslant 0$ and $L_{ij} \leqslant 0$ for $j \neq i$. Assume moreover that $L_{ii} \geqslant \sum_{j \neq i} |L_{ij}|$ and that L is irreducible. The determinant of L then vanishes if and only if $\sum_{j=1}^{N} L_{ij} = 0$ for $i = 1, 2, ..., N$.

As the determinant does not vanish, the zero eigenvalue of the Laplacian disappears and the Gershgorin circle theorem ensures that all the eigenvalues of $-L^*$ have then negative real part. □

S3 Examples of Graphs Illustrating the Characteristics of the First Left Eigenvector

Example S1. *Consider the digraph in Table S1 with its Laplacian matrix and its first left eigenvector. Lemma S2 can be illustrated through this example.*
Nodes 2,3, and 4 are not globally reachable (they cannot be reached from node 1), but still they have both incoming and outgoing edges. The second, third and fourth entries of the first left eigenvector are then zeros.

Table S1. Graph 1 example

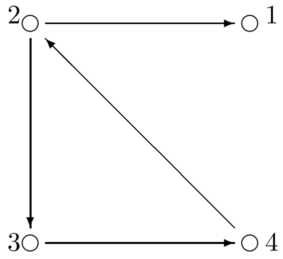

$$L = \begin{bmatrix} 0 & 0 & 0 & 0 \\ -1 & 2 & -1 & 0 \\ 0 & 0 & 1 & -1 \\ -1 & 0 & 0 & 1 \end{bmatrix} \quad \mathbf{v} = \begin{Bmatrix} 1 \\ 0 \\ 0 \\ 0 \end{Bmatrix}$$

Example S2. *Consider the line digraph in Table S2 with its Laplacian matrix and its first left eigenvector. Lemma S4 can be explained through this example.*

Table S2. Graph 2 example

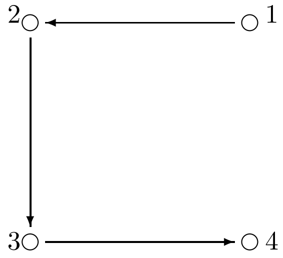

$$L = \begin{bmatrix} 1 & -1 & 0 & 0 \\ 0 & 1 & -1 & 0 \\ 0 & 0 & 1 & -1 \\ 0 & 0 & 0 & 0 \end{bmatrix} \quad \mathbf{v} = \begin{Bmatrix} 0 \\ 0 \\ 0 \\ 1 \end{Bmatrix}$$

Consider, in particular, the second entry of the FLE: it is null. According to Lemma S4, this corresponds to a nonzero diagonal entry L_{22}, which is actually verified here. The only way to have $L_{22} = 0$ would be dropping the 2-3 edge. Because the only incoming connection to node 2 has origin in a node that is not globally reachable, the resulting graph would be disconnected.

S4 Further Bounds to the Convergence Speed

Using the triangle inequality, the convergence speed can be bounded. Consider Equation (6), it holds

$$\|\mathbf{v}\|_2 \leq \|\mathbf{v}\|_1. \tag{S4}$$

In the case $\|\mathbf{v}\|_2 \geq 1$ the smallest eigenvalue in magnitude of the perturbed Laplacian can also be related to the L_1 norm of the first left eigenvector, being bounded by

$$\lambda_1(-L - C) \geq -\frac{\|\mathbf{v}\|_1^2}{\alpha}. \tag{S5}$$

Finally, when the L_1 norm of $\mathbf{v}$ is unitary, Eq. (S5) reduces to

$$\lambda_1(-L - C) \geq -1. \tag{S6}$$

S5 First Left Eigenvector with Frobenius Matrix Norm

For the particular case of a Frobenius matrix norm for the perturbation, the same arguments used to find the diagonal matrix that maximizes the spectral radius in [HNT99] can be adopted to find the minimum

of it. Consider the gradient of the spectral radius in Eq. (10) and consider the eigenprojection of the generic matrix A as defined in [AC02]. It can be stated that the gradient in Eq. (10) corresponds to the transpose of the eigenprojection E, that is

$$E = \frac{\mathbf{u}\mathbf{v}^T}{\mathbf{v}^\mathbf{T}\mathbf{u}} \tag{S7}$$

$$\nabla\rho(A) = E^T = \frac{\mathbf{v}\mathbf{u}^T}{\mathbf{v}^\mathbf{T}\mathbf{u}}. \tag{S8}$$

A diagonal perturbation E_D composed of the Perron vector attains the minimum of the spectral radius for the matrix $(-L - E_D + kI)$ amongst the diagonal perturbations with fixed Frobenius norm, in the hypothesis of small magnitude of the perturbation. This is shown through the following lemma.

Lemma S5. *Let L be the Laplacian matrix of a directed graph on N nodes. Let $\mathbf{u}$ and $\mathbf{v}$ be respectively the right and left Perron vectors associated with the zero eigenvalue. Let k be a positive scalar greater than the largest eigenvalue in magnitude of L and E_D be the diagonal matrix defined as*

$$E_D = \operatorname{diag}\left(\frac{\mathbf{u} \circ \mathbf{v}}{\mathbf{v}^\mathbf{T}\mathbf{u}}\right) \tag{S9}$$

where $\{\circ\}$ indicates the product element by element. Then the minimum of the spectral radius of $(-L - \Delta + kI)$ with Δ belonging to the space of diagonal matrices of unitary Frobenius norm is achieved for $\Delta = E_D/\|E_D\|_F$.

Proof. Consider the derivative of the spectral radius of the matrix $M = -L + kI$ in the direction of Δ and $E_D/\|E_D\|_F$, respectively

$$\begin{aligned}\rho'_\Delta(M) &= \lim_{t\to 0} \frac{\rho(M - t\Delta) - \rho(M)}{t} \\ &= -E^T \cdot \Delta = -trace(E\Delta) \qquad \text{(S10)}\\ \rho'_{E_D/\|E_D\|_F}(M) &= \lim_{t\to 0} \frac{\rho\left(M - t\frac{E_D}{\|E_D\|_F}\right) - \rho(M)}{t} \\ &= -E^T \cdot \frac{E_D}{\|E_D\|_F} = -\|E_D\|_F. \qquad \text{(S11)}\end{aligned}$$

The inner product between matrices is defined as

$$P \cdot Q = trace\left(P^T Q\right);$$

which provides a definition for the Frobenius norm as $\|P\|_F = \sqrt{trace\,(P^T P)}$. For the *Cauchy-Schwarz* inequality

$$trace(E\Delta) = E^T \cdot \Delta \leq \|E^T\|_F \|\Delta\|_F = \|E\|_F, \tag{S12}$$

which implies

$$\rho'_{E_D/\|E_D\|_F}(M) \leq \rho'_\Delta(M). \tag{S13}$$

□

Because of how E_D is defined in Eq. (S9) and because of the fact that M has the same eigenvectors as L, $\mathbf{u}$ is always uniform and the gradient of the spectral radius of M depends upon the left eigenvector only.

S6 Second Order Dynamics, Existence of the Zero Eigenvalue

For positive C^d, C^v and C^w the system matrix in Eq.(16) is not Hurwitz as it keeps the zero eigenvalue, as proved in Lemma S6.

Lemma S6. *Let L be the Laplacian matrix of a connected directed graph on N nodes. Let C^d, C^v and C^w be diagonal matrices with nonnegative entries and in particular let C^w have nonzero elements along the diagonal where the first left eigenvector, corresponding to the zero eigenvalue of the Laplacian matrix, presents nonzero entries. Then 0 is an eigenvalue of the matrix*

$$S = \begin{bmatrix} [0] & I \\ -C^d L & -(C^v L + C^w) \end{bmatrix}. \tag{S14}$$

Proof. The existence of the 0 eigenvalue is proved by proving the determinant to be null. Consider a generic matrix M partitioned as

$$M = \begin{bmatrix} A & B \\ C & D \end{bmatrix}. \tag{S15}$$

According to the Schur formula, the determinant of the generic matrix M is

$$\mathrm{det(M) = |M| = |AD - BD^{-1}CD|}. \tag{S16}$$

Likewise, the determinant of matrix S is

$$\begin{aligned} \mathrm{det(S)} &= |S| = |[0] - (C^v L + C^w)^{-1}(-C^d L)(C^v L + C^w)| = \\ &= |(C^v L + C^w)^{-1}||C^d L||C^v L + C^w| = |C^d L| = 0. \end{aligned} \tag{S17}$$

□

S7 Second Order Dynamics, Conjectured Stability

Proving the existence of the zero eigenvalue in the spectrum of S is not enough to conclude the system is stable. To achieve this, it should be proved, for instance, that all the eigenvalues of matrix S have nonpositive real part. This is not the case for all possible values of C^d and C^v as numerical simulations show. However, for a wide range of values and graphs the system appears stable. The stability of matrix S is hence here conjectured and supported by some complementary findings and numerical analysis.

Conjecture 1. *The second order dynamical system whose state space representation is described by matrix* (S14) *is stable for small values of the vector C_i^d for $i = 1, 2..., N$.*

The conjecture is supported by the following statements:

- The trace of the matrix (S14) is negative. This corresponds to the sum and the average of the eigenvalues being negative.

- If the graph is undirected, the Laplacian would be symmetric and so would the matrices $C^v L + C^w$ and $-C^d L$. The matrix (S14) then would describe a system of interconnected oscillators with a positive semidefinite stiffness matrix and a positive definite damping matrix. Note that in the symmetric case, having all the eigenvalues positive (resp. nonnegative) is a sufficient condition to conclude the matrices are positive definite (resp. semidefinite). In this particular case the stability does not depend upon the value of C^d.

- For the case of a 2 node graph, it can be easily verified that the signs of the characteristic polynomial do not change. Then Descartes' rule of sign can be invoked and, together with the trace being negative, this indicates all the eigenvalues are nonpositive. The 2 node graph case is detailed in the following. As for the symmetric case, also this time the stability does not depend upon the value of C^d.

- In line with the findings in [Sha05], the stability in the case of a generic, directed graph, appears dependent on the degree of asymmetry of the stiffness matrix. In particular, in [Sha05] it is shown how, for a symmetric lower-right partition ($C^vL + C^w$ for our system) of the system matrix, the stability of the system can be linked to the amount of asymmetry present in the lower-left partition ($-C^dL$ for our system) compared to its symmetric part and to the lower-right partition. However here, both the lower partitions of the matrix are non symmetric, and the relative magnitude of the two differ because of the C^w, C^d and C^v coefficients. As C^w is here determined by the first left eigenvector of the Laplacian matrix, the value of C^d regulates the stability of the system. As the value of C^d decreases the matrix partition $C^vL + C^w$ becomes more dominant with respect to $-C^dL$, producing a more stable behaviour, as observed in [Sha05].

Conjecture 1 is furthermore supported by the numerical tests reported in the Results section and complemented by the other cases in Fig. S3 and S4.

The 2-node graph case for the second order system stability

Consider a matrix describing the second order system in state space as in Eq. (S14). In particular consider the case of a 2 node graph as in Eq. (S18)

$$S_2 = \begin{bmatrix} 0 & 0 & 1 & 0 \\ 0 & 0 & 0 & 1 \\ -C_1^d L_{11} & -C_1^d L_{12} & -C_1^u - C_1^v L_{11} & -C_1^v L_{12} \\ -C_2^d L_{21} & -C_2^d L_{22} & -C_2^v L_{21} & -C_2^u - C_2^v L_{22} \end{bmatrix}. \tag{S18}$$

The characteristic polynomial can be developed as

$$\begin{aligned} \det(\mathrm{S}_2 - \lambda \mathrm{I}) \quad = \quad & \lambda^4 + (C_1^w + C_2^w + C_1^v L_{11} + C_2^v L_{22})\lambda^3 \\ & + (C_1^w C_2^w + C_1^d L_{11} + C_2^d L_{22} + C_2^w C_1^v L_{11} + C_1^w C_2^v L_{22} + C_1^v C_2^v L_{11} L_{22} - C_1^v C_2^v L_{12} L_{21})\lambda^2 \\ & + (C_1^d C_2^w L_{11} + C_2^d C_1^w L_{22} + C_1^v C_2^d L_{11} L_{22} - C_1^v C_2^d L_{12} L_{21} + C_2^d C_1^v L_{11} L_{22} - C_2^d C_1^v L_{12} L_{21})\lambda \\ & + C_1^d C_2^d L_{11} L_{22} - C_1^d C_2^d L_{12} L_{21}. \end{aligned} \tag{S19}$$

The constant term in Eq. (S19) is null as it is the determinant of C^dL, that is

$$C_1^d C_2^d L_{11} L_{22} - C_1^d C_2^d L_{12} L_{21} = |C^d L| = 0.$$

Moreover all the coefficients of the characteristic polynomial are positive. It is sufficient to note that the entries of C^w, C^v and C^d are non-negative while $L_{11}, L_{22} \geq 0$ and $L_{12}, L_{21} \leq 0$. Thus every term in the characteristic polynomial is nonnegative, and within each coefficient some term must be positive. For Descartes' sign rule all the nonzero roots of the characteristic polynomial have the same sign. As the trace of matrix S_2 is negative, none of the roots of the characteristic polynomial can be positive.

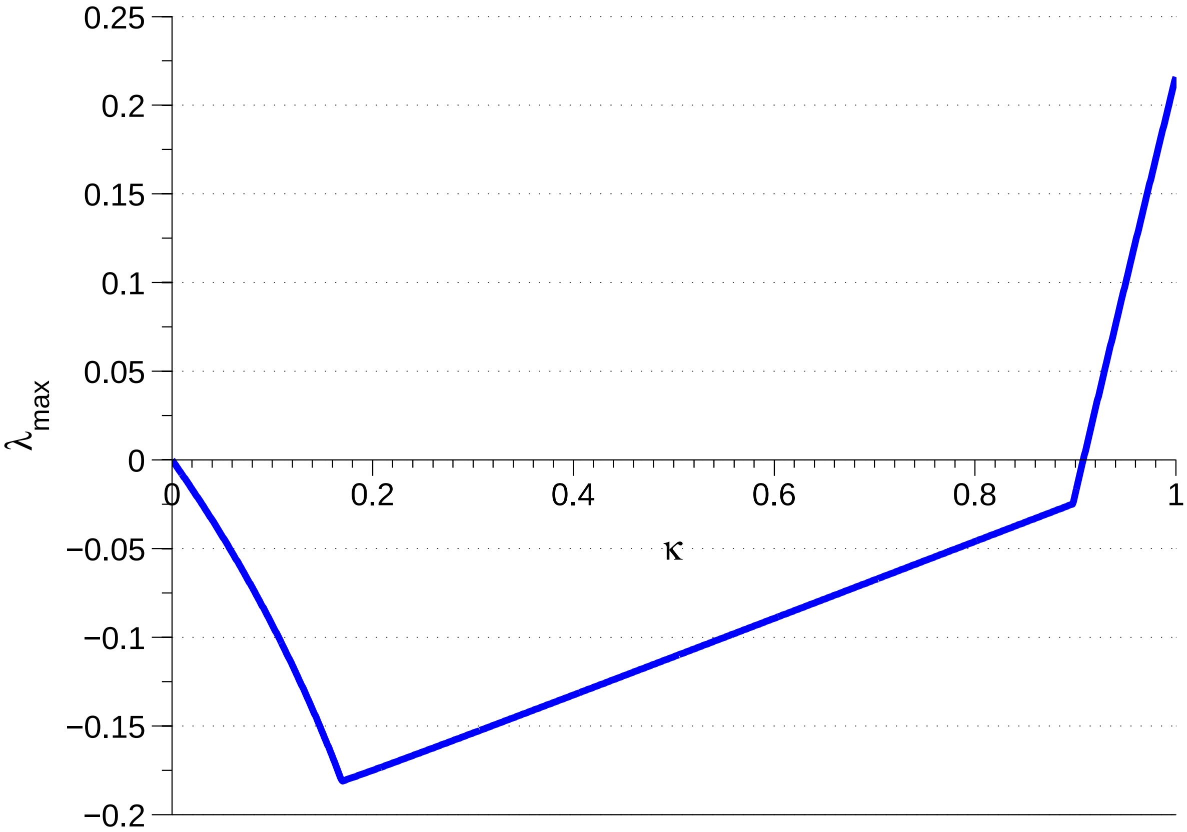

Figure S1. Largest eigenvalue (considering the sign) of the system matrix as a function of the free parameter κ for the asymmetric lattice in Fig. 1.a.

S8 Numerical Analysis of the Stability Limits

For the case of a connected and directed graph, the role of the C^d and C^v parameters is here analysed through numerical means. The asymmetric lattice used in the first order dynamics numerical examples is here used to show the dependence of the stability on the asymmetry in the system matrix. In accordance with the condition (14), C_i^d and C_i^v for each node i are defined as

$$C_i^d = \kappa(1 - C_i^w) \quad and \quad C_i^v = (1-\kappa)(1 - C_i^w) \tag{S20}$$

using the free parameter $\kappa \in [0, 1]$. The value of the largest nonzero eigenvalue (not just the magnitude but also the sign is considered) for 1000 values of the free parameter κ are plotted in Fig. S1. At $\kappa = 0.9075 \pm 0.0005$ the largest eigenvalue changes sign making the second order system unstable.
The appearance of positive eigenvalues meets the criterion established in [Sha05] where instability is linked, for a particular class of systems, to the degree of asymmetry of the lower left partition, C^dL in this case. To confirm this, the matrix norms of the partitions are plotted in Fig. S2, where it is shown how, by increasing the parameter κ, the skew-symmetric part of matrix C^dL increases linearly, matching and surpassing the norm of the $C^vL + C^w$ matrix. For a generic matrix M, the matrix norm is defined as

$$\|M\|, \triangleq max\{\|M\mathbf{v}\|\} = \sqrt{\lambda_{max}\{M^*M\}} \tag{S21}$$

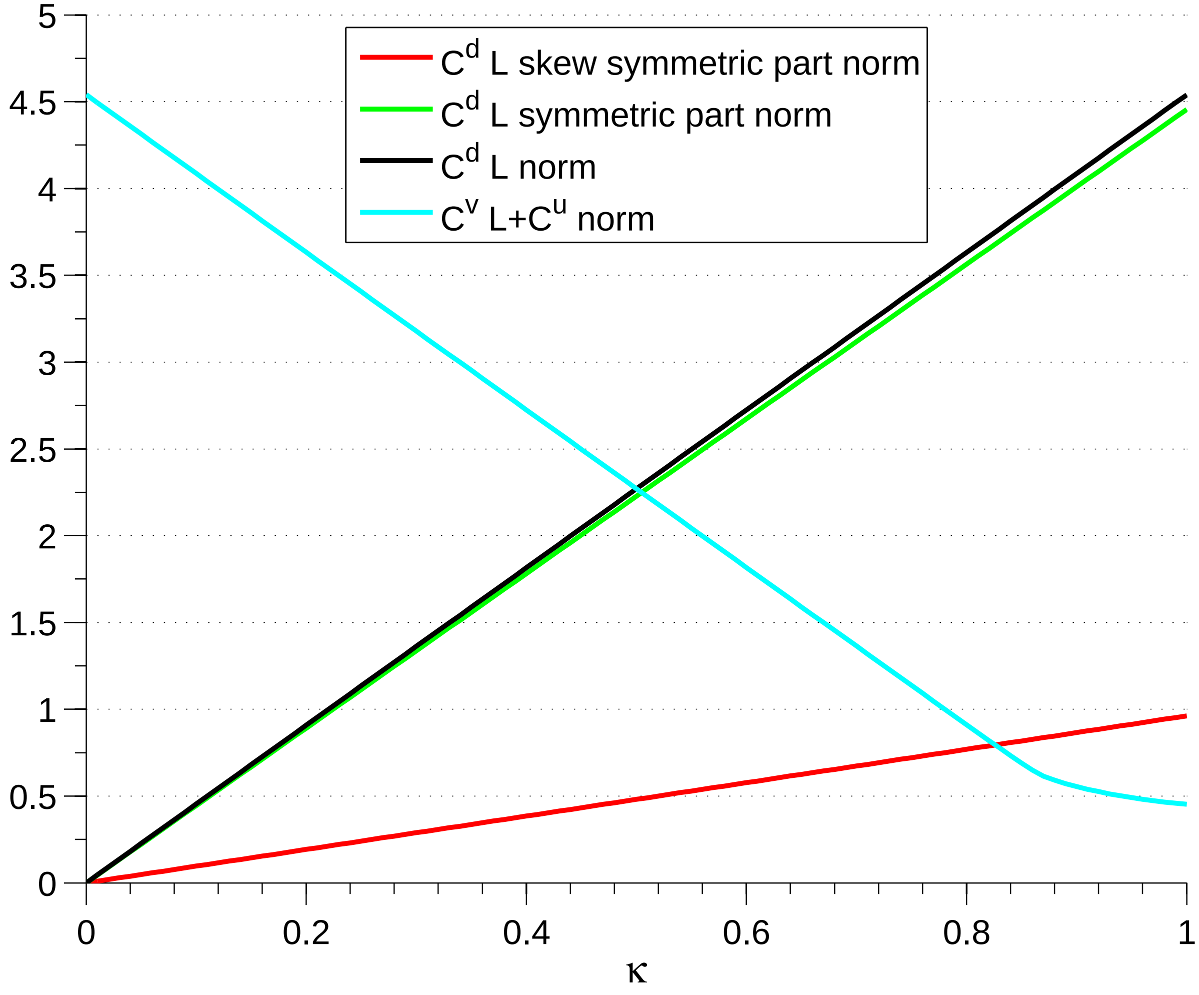

Figure S2. Norms of the system matrix partitions as a function of the free parameter κ.

for all possible vectors $\mathbf{v}$ of unitary norm, where M^* denotes the conjugate transpose of M.
The findings in [Sha05] are not directly applicable to this case, because they refer to matrices with a symmetric lower right partition and because they just provide necessary conditions for stability. However, also here, within the limits of the numerical analysis, an increase in the asymmetry of the lower left partition with respect to the lower right corresponds to the increase of instability in the system.

S9 Second Order Dynamics, Consensus in a Lattice Network for $\kappa = 0.5$ and $\kappa = 0.8$

The value of the parameter κ determines the amount of resources each agent allocates to the agreement about a common velocity and a common separation with respect to its neighbours. A high value of the parameter κ destabilises the system. Here the dynamics of the second order system for $\kappa = 0.5$ and $\kappa = 0.8$ are shown in Fig. S3 and S4 to complement what was claimed in the main part of this work.

The figures show how the system becomes less and less stable as κ increases towards unity.

S10 Fastest Convergence with Fixed Trace Diagonal Perturbation

For a Laplacian perturbed by a diagonal perturbation of fixed L_1 norm, Eq. 6 indicates the FLE maximises the consensus speed. However, it was noted that limiting L_1 norm of the perturbation does not corresponds to requiring a fixed trace diagonal perturbation. The following theorem and lemma, from [JSOvdD94], provide a useful means to find the perturbation matrix T, in the space of diagonal matrices with fixed trace that maximises the consensus speed of the perturbed Laplacian. In particular the theorem provides a way to calculate the matrix T with zero trace which returns the lowest spectral radius possible and the lemma extends the results to all possible traces. Note that when elements of T are all nonnegative, the trace corresponds to the L_1 norm of the vector composed by the same nonzero entries of T. To relate the following theorem and lemma to the results presented here, the following notation is needed:

- $\mu(A;t)$ is the minimum of the spectral radius for a generic matrix A subject to a diagonal perturbation with trace t. In particular for zero trace perturbation, let $\mu(A) \triangleq \mu(A;0)$.
- T_q indicates a diagonal matrix whose nonzero entries are the elements of vector $\mathbf{q}$.
- The diagonal similarity of a matrix A is defined as $A_1 = T_q^{-1}AT_q$ where $\mathbf{q}$ is a positive vector. The diagonal similarity of a nonnegative matrix A is nonnegative too and the value of $\mu(A)$ is not affected by the transformation.

Theorem S3. *Let A be an $N \times N$ essentially nonnegative matrix, and suppose that P is a permutation matrix such that P^TAP is in Frobenius normal form. Let A_0 be the direct sum of irreducible matrices that is obtained from P^TAP by replacing all entries in off-diagonal blocks with 0's, and let B be the line sum symmetric diagonal similarity of A_0. Then $\mu(A) = \mu(A_0) = (1/N)\mathbf{1}^TB\mathbf{1}$, and $\mu(A) = min\{\lambda_{max}(A+T_q) : \mathbf{1}^T\mathbf{q} = 0\}$ is achieved only by $\mathbf{q} = P[\mu(A)\mathbf{1} - B\mathbf{1}]$.*

Lemma S7. *Let A be an $N \times N$ essentially nonnegative matrix and let t be a scalar. Then $\mu(A;t) = \mu(A) + t/N$. Furthermore if the minimum $\mu(A)$ is achieved only by the vector $\mathbf{q}$ with $\mathbf{1}^T\mathbf{q} = 0$, then the minimum $\mu(A;t)$ is achieved only by a vector $\mathbf{q} + t/N\mathbf{1}$.*

An important consideration is that the calculation of the matrix B from Theorem S3 is not immediate. It consists in finding the positive diagonal matrix T_q that makes matrix A_0 line sum symmetric. This corresponds to finding the solution of the system of equations

$$T_q^{-1}A\mathbf{q} - T_qA^T\delta = \mathbf{0} \tag{S22}$$

where δ is a vector whose elements are the inverse of the elements of $\mathbf{q}$. This can be solved through iterative procedures such as the Newton-Raphson method. The above theorem and lemma provide a useful way to calculate the best diagonal perturbation for fixed trace. Unfortunately the fixed trace condition produces, in many cases, the effect of "fouling" the less influential nodes to allocate more influence to the most influential ones. As the arithmetic sum of the diagonal elements must be the same, some elements (those corresponding to less observed nodes) can be taken negative so as to allow other elements to achieve larger values, even beyond the total amount available. Assigning negative values does not match the initial intent of distributing resources in an efficient way as a negative element, that corresponds to passing a node wrong information about the driving signal, requires a resource allocation by the node anyway. The L_1 norm in this case would exceed the trace, representing the actual maximum amount of resources assigned overall.

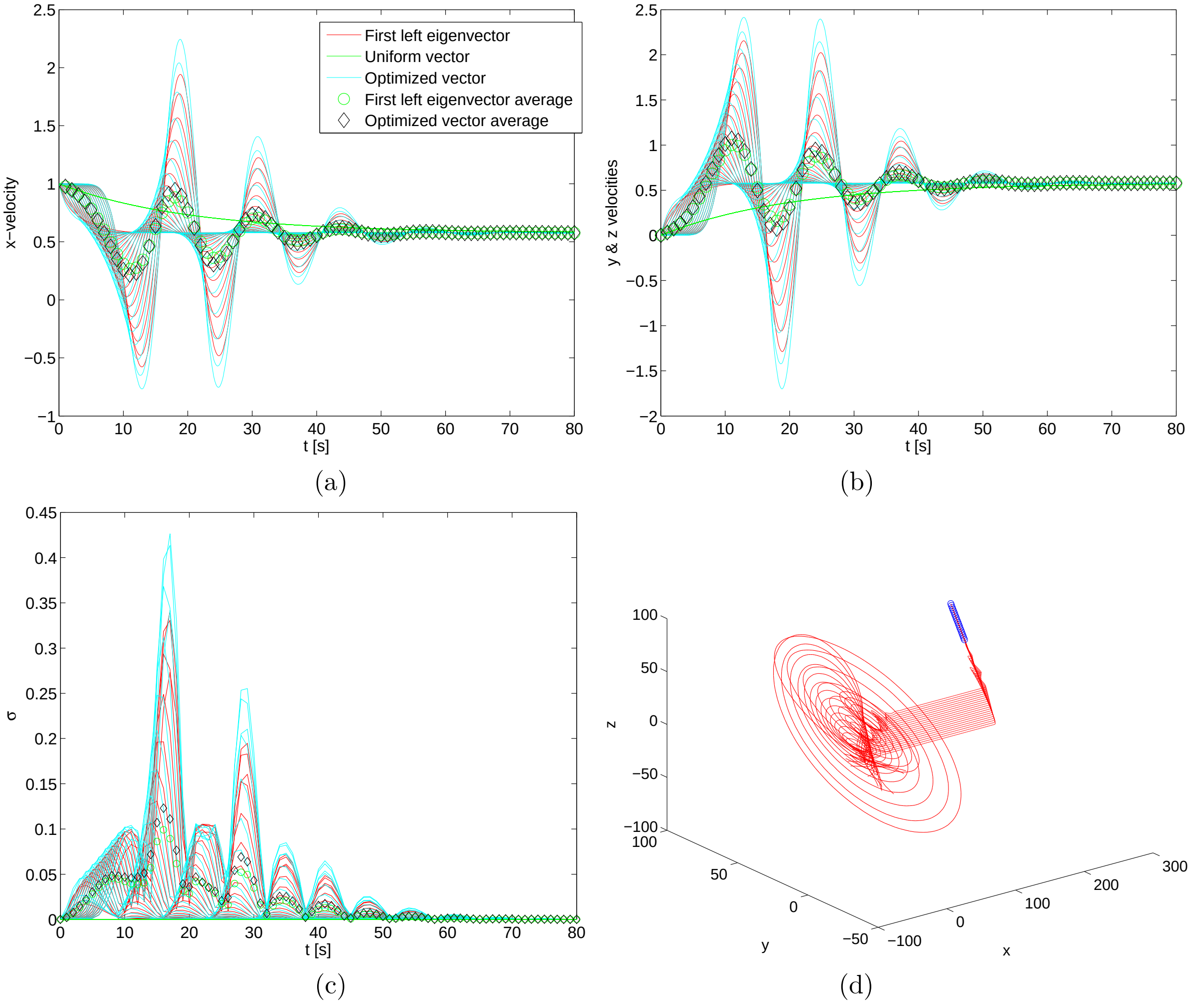

Figure S3. Consensus in a lattice network, for a second order system for $\kappa = 0.5$. (a) Consensus about x velocity. (b) Consensus about y and z velocity. (c) Standard deviations of the agent relative distances over time showing consensus about common reciprocal separation. (d) Trajectories in physical space of the agents from the initial random state to consensus about a common direction and relative distances. At $\kappa = 0.5$ oscillations become more evident.

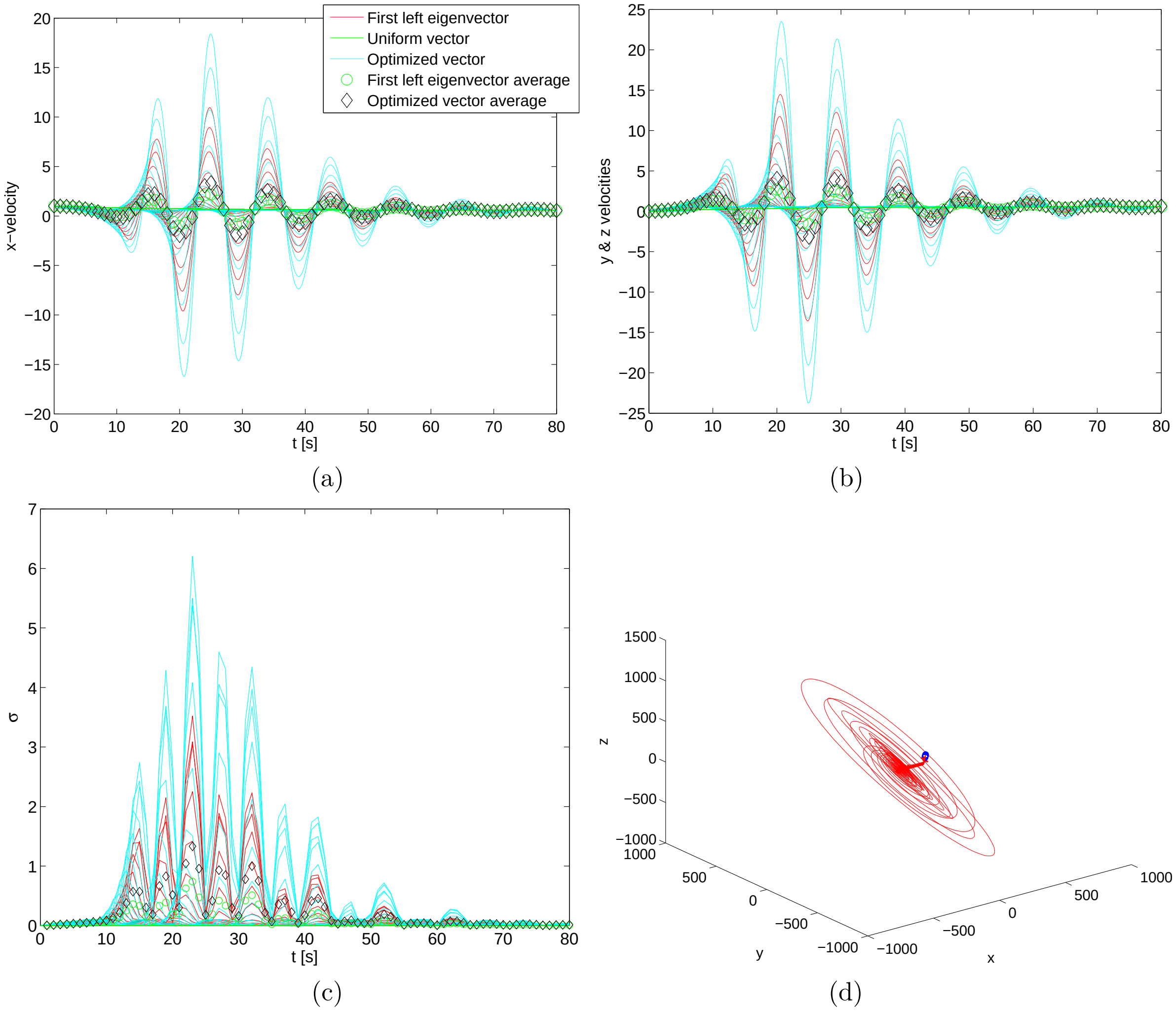

Figure S4. Consensus in a lattice network, for a second order system for $\kappa = 0.8$. (a) Consensus about x velocity. (b) Consensus about y and z velocity. (c) Standard deviations of the agent relative distances over time showing consensus about common reciprocal separation. (d) Trajectories in physical space of the agents from the initial random state to consensus about a common direction and relative distances. At $\kappa = 0.8$ oscillations are close to being disruptive of the system.

References

AC02. Agaev RP, Chebotarev PY On determining the eigenprojection and components of a matrix. *Autom. Remote Control*, 63(10):1537–1545, October 2002.

HNT99. Han L, Neumann M, Tsatsomeros M. Spectral radii of fixed frobenius norm perturbations of nonnegative matrices. *SIAM J. Matrix Anal. Appl.*, 21(1):79–92, October 1999.

JSOvdD94. Johnson CR, Stanford DP, Dale Olesky D, and van den Driessche P. Dominant eigenvalues under trace-preserving diagonal perturbations. *Linear Algebra and Its Applications*, 212-213:415–435, 1994.

Sha05. Shapiro A. Stability of second-order asymmetric linear mechanical systems with application to robot grasping. *Journal of Applied Mechanics*, 72(6):966–968, 2005. 10.1115/1.2042484.

Tau49. Taussky O. A recurring theorem on determinants. *The American Mathematical Monthly*, 56(10):pp. 672–676, 1949.
